# Use of live attenuated recombinant Newcastle disease virus carrying avian paramyxovirus 2 HN and F protein genes to enhance immune responses against species A rotavirus VP6 protein

**DOI:** 10.1186/s13567-024-01271-4

**Published:** 2024-02-05

**Authors:** Rofaida Mostafa Soliman, Keisuke Nishioka, Fumi Murakoshi, Takaaki Nakaya

**Affiliations:** 1 Department of Infectious Diseases, Kyoto Prefectural University of Medicine, Kyoto, Japan; 2 Department of Animal Medicine (Infectious Diseases Division), Faculty of Veterinary Medicine, Damanhour University, Damanhour, El‑Beheira Egypt; 3 Laboratory of Sustainable Animal Environment, Graduate School of Agricultural Science, Tohoku University, Miyagi, Japan; 4 Frontier Research Institute for Interdisciplinary Sciences, Tohoku University, Miyagi, Japan

**Keywords:** Newcastle disease virus vector, bovine rotavirus, vaccine, chimeric virus, immune responses

## Abstract

Numerous infectious diseases in cattle lead to reductions in body weight, milk production, and reproductive performance. Cattle are primarily vaccinated using inactivated vaccines due to their increased safety. However, inactivated vaccines generally result in weaker immunity compared with live attenuated vaccines, which may be insufficient in certain cases. Over the last few decades, there has been extensive research on the use of the Newcastle disease virus (NDV) as a live vaccine vector for economically significant livestock diseases. A single vaccination dose of NDV can sufficiently induce immunity; therefore, a booster vaccination dose is expected to yield limited induction of further immune response. We previously developed recombinant chimeric NDV (rNDV-2F2HN), in which its hemagglutinin-neuraminidase (HN) and fusion (F) proteins were replaced with those of avian paramyxovirus 2 (APMV-2). In vitro analysis revealed that rNDV-2F2HN expressing human interferon-gamma had potential as a cancer therapeutic tool, particularly for immunized individuals. In the present study, we constructed rNDV-2F2HN expressing the bovine rotavirus antigen VP6 (rNDV-2F2HN-VP6) and evaluated its immune response in mice previously immunized with NDV. Mice primarily inoculated with recombinant wild-type NDV expressing VP6 (rNDV-WT-VP6), followed by a booster inoculation of rNDV-2F2HN-VP6, showed a significantly stronger immune response than that in mice that received rNDV-WT-VP6 as both primary and booster inoculations. Therefore, our findings suggest that robust immunity could be obtained from the effects of chimeric rNDV-2F2HN expressing the same or a different antigen of a particular pathogen as a live attenuated vaccine vector.

**Supplementary Information:**

The online version contains supplementary material available at 10.1186/s13567-024-01271-4.

## Introduction

Emerging and re-emerging infectious diseases are a cause of concern in public health as well as in the livestock industry [1]. There are frequent epidemics in the livestock sector, which lead to substantial economic losses and require prompt countermeasures at the farm level. In cattle, infectious disease outbreaks can lead to reductions in body weight, milk production, and reproductive performance. Accordingly, vaccination represents a crucial countermeasure in this regard.

Inactivated vaccines are being extensively used for various infectious diseases in cattle, especially pregnant animals, allowing for the transfer of passive immunity to the offspring through colostrum during the neonatal period [2]. Inactivated vaccines can be administered as monovalent or bivalent formulations and sometimes even as multivalent blends with other antigens. However, the effectiveness of these vaccines can be compromised due to factors such as insufficient maternal immunity, mixed infections, and encounters with reassortant viruses [3]. The resulting inadequate immune responses may necessitate multiple rounds of vaccination. By contrast, live attenuated vaccines are robust inducers of immunity and can often establish long-term immunological memory with a single dose [4]. However, concerns about their contribution to genetic divergence and low safety, particularly in pregnant animals, limit their widespread use [5–7]. Therefore, there is a pressing need for safe and effective vaccine vectors capable of targeting multiple pathogens in bovines [8].

Newcastle disease virus (NDV) poses a high pathogenic threat to poultry, and live attenuated vaccines have been developed for use in chicken [9]. Additionally, NDV has been extensively applied as a vaccine vector and an anticancer therapeutic tool owing to its unique oncolytic properties [10–15]. The recombinant NDV (rNDV) vector has been developed to express various foreign genes for a wide range of applications [14, 16–19]. For example, an rNDV expressing foreign antigens such as hemagglutinin (HA) of the influenza virus has been shown to elicit immunity against HA [12, 20, 21]. Furthermore, NDV has a broad range of hosts, including humans, mice, cats, dogs, and cattle; therefore, it is an attractive vaccine vector for diverse animal pathogens, including bovine-specific pathogens [10, 17, 20, 22, 23]. However, homologous vaccines administered through primary and booster doses might have limited efficacy in generating robust immune responses. To address this issue, recombinant chimeric NDVs have been engineered through the replacement of the F or HN protein with APMV-2 or the incorporation of surface proteins comprising portions of both NDV and APMV-2 polypeptides [24–26]. Administration of chimeric NDVs expressing the same or different antigens during booster inoculation may be a promising strategy for fostering the development of potent immunity.

Group A bovine rotavirus (BRV) stands as a significant contributor to severe calf diarrhea worldwide, often resulting in notable economic repercussions due to treatment expenses, decreased growth rates, and decreased overall productivity [27, 28]. In light of this, we generated recombinant NDVs encompassing both wild-type (WT) and chimeric viruses, that contain the BRV VP4 or VP6 antigens, and we assessed their ability to induce immune responses in mice.

## Materials and methods

### Cells and rotaviruses

Human respiratory epithelial cells (HEp-2 cells), chicken embryo fibroblasts (CEF cells), bovine intestinal epithelial cells (BIE cells) [29, 30], Madin-Darby bovine kidney cells (MDBK cells), and African green monkey kidney cells (MA104 cells) were maintained in a medium supplemented with 10% fetal bovine serum, 100 U/mL penicillin, and 100 μg/mL streptomycin. Bovine rotavirus (BRV Lincoln strain (G6, P[1])) and simian rotavirus (SA11 strain (G5, P[3])) (ATCC® VR-1565™) were propagated in the MA104 cell line as previously described [31]. The viral titers were determined using the fluorescence focus units (FFU) as previously described [32]. All virus stocks were stored at -80 °C until use. The BRVs were activated using trypsin (Sigma Aldrich, MO, USA) stocked at 1 mg/mL and were diluted to a final concentration of 10 μg/mL, added to viral stock vials, and incubated for 30 min at 37 °C before in vitro infection. All experiments were performed at the Kyoto Prefectural University of Medicine under the Biosafety Level 2 conditions.

### Recombinant chimeric NDV genome construction and rescue experiment

**Figure 1:**
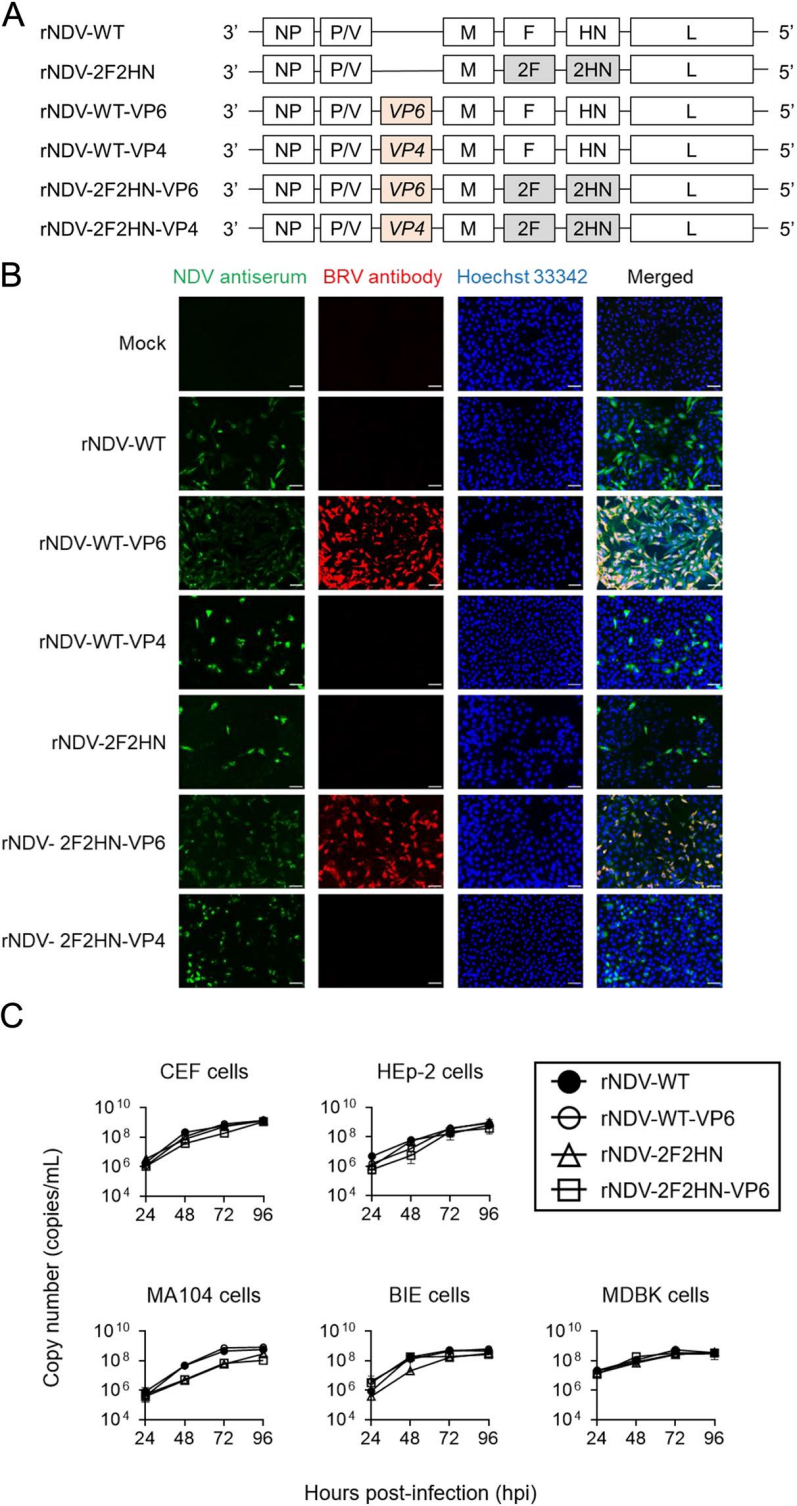
**Construction of recombinant NDVs expressing the VP6 gene of BRV.** **A** Schema of constructed recombinant NDV genomes inserted with the BRV VP6 or VP4 gene. **B** Immunostaining of HEp-2 cells infected with each recombinant NDV. The infected cells were stained using anti-NDV antiserum (green), BRV antibody (red), and Hoechst 33342 (blue; nuclei). Scale bar, 50 µm. **C** Growth kinetics of recombinant viruses in different cells. The cells were infected at MOI of 0.1 for each of the viruses. The copy number from the culture supernatants was determined using real-time PCR at 24-h intervals.

BRV VP4 and VP6 (accession no. AB119636.1 and JF693031.1) were amplified by reverse transcription polymerase chain reaction (RT-PCR) using specific primer sets (Additional files 5 and 6); subsequently, they were inserted between the P and M genes of the rNDV-WT or rNDV-2F2HN cDNA clones. Six recombinant cDNA with or without VP4 and VP6 were generated (Figure 1A); additionally, recombinant viruses were obtained as previously described [12]. The pathogenicity of the recombinant viruses in poultry was determined by the mean death time (MDT) assay using embryonated chicken eggs [33].

To evaluate the genetic stability of VP6 within the rNDVs, the recovered viruses were serially passaged five times in MDBK cells. After the five passages, the presence and expression of VP6 were confirmed by RT-PCR and immunofluorescence (Additional file 2).

### Immunofluorescence assay (IFA)

HEp-2 cells were cultured in a 96-well plate (1.0 × 10^4^ cells/well) for 24 h and infected with rNDVs at a multiplicity of infection (MOI) of 0.5 [34]. On the next day, the cells were fixed with 4% paraformaldehyde in phosphate-buffered saline (PBS) for 10 min and permeabilized with 0.2% Triton X-100 in PBS for 10 min at room temperature. Chicken anti-NDV antisera and goat anti-BRV polyclonal antibodies (cat. ab20036; Abcam, Cambridge, UK) were used as primary antibodies for detecting NDV and BRV antigens, respectively. The secondary antibodies used were goat anti-chicken IgY-conjugated Alexa-488 (cat. A11039, Thermo Fisher Scientific, Waltham, MA, USA), donkey anti-goat IgG conjugated Alexa-555 (cat no. AP180C; Thermo Fisher Scientific) and goat anti-mouse IgG conjugated Alexa-488 (cat no. A11029 Thermo Fisher Scientific). Hoechst 33342 (DOJINDO, Kumamoto, Japan) was used for nucleus staining. The infected cells were observed under fluorescence microscopy BZ-X700 (KEYENCE, Osaka, Japan).

### Viral replication assay

HEp-2, CEF, MA104, MDBK, and BIE cells were cultured in 24-well plates (1.0 × 10^5^ cells/well) and infected with the rNDVs at an MOI of 0.1. The supernatant was collected at 24 h intervals until 96 h after infection. Total RNA was extracted from each sample using ISOGEN II (Nippon Gene, Tokyo, Japan); additionally, reverse transcription was performed using the ReverTra Ace qPCR RT Master Mix (TOYOBO, Osaka, Japan). The viral copy number was determined by real-time PCR using PowerUp SYBR Green Master Mix (Thermo Fisher Scientific, Waltham, MA, USA) and the following forward (5′-AGTGATGTGCTCGGGCCTTC-3′) and reverse (5′-CCTGAGGAGAAGCATTTGCTA-3′) primers as previously described [26].

### Animal experiments

To assess the pathogenicity of the recombinant viruses, 4-week-old female BALB/c mice were inoculated with 1 × 10^7^ FFU of each virus via the intranasal or intraperitoneal routes. All mice were monitored daily for signs of illness, weight change, or death for 2 weeks.

To evaluate immune responses against BRV VP6, ten experimental groups (inoculation combination) were prepared as follows: (1) and (6) rNDV-WT and rNDV-WT, (2) and (7) rNDV-WT and rNDV-2F2HN, (3) and (8) rNDV-WT-VP6 and rNDV-WT-VP6, (4) and (9) rNDV-WT-VP6 and rNDV-2F2HN-VP6, and (5) and (10) PBS and PBS. The mice groups (1)–(5) and (6)–(10) were inoculated intraperitoneally and intranasally, respectively. The 4-week-old female BALB/c mice were inoculated with 1 × 10^7^ FFU of rNDVs, while the control group received PBS. Three weeks after the primary inoculation, the mice were boosted with rNDVs. The blood samples from the different groups were collected twice (3 weeks after primary and 3 weeks after booster inoculations), and the serum was separated for serological assays. Three weeks after booster inoculations, the mouse splenocytes were harvested as previously reported [35] and stored in Bambanker (NIPPON Genetics, Tokyo, Japan) at −80 °C until use.

### Neutralization assay

The sera of immunized mice were serially two-fold diluted and mixed with an equal amount of 100 FFU of BRV or 1000 FFU of NDV solution and incubated at 37 °C for 1 h. The mixture was added to the target cells in triplicate wells of a 96-well plate. At 24 post-infection hours, an immunofluorescence assay was performed, and the infected cells were counted using a fluorescence microscope. The neutralizing titers were calculated as the BRV infection inhibition ratio or the highest dilution ratio that completely inhibited the NDV infection.

### Splenocyte proliferation assay

To detect the antigen-specific responses in mouse splenocytes, the cell proliferation after stimulation with each antigen was assessed. The splenocytes were seeded in 96-well plates (1 × 10^5^ /well). Two days after stimulation, cell viability was measured using the Cell Count Reagent SF (Nacalai Tesque, Kyoto, Japan).

### Statistical analysis

Data are expressed as the arithmetic mean ± standard error of the mean (SEM). All statistical analyses were performed using Student’s *t*-test with the GraphPad Prism 8 software (La Jolla, CA, USA). Statistical significance was set at *P* < 0.05.

## Results

### BRV-VP6 antigen was expressed in infected cells with recombinant NDVs

We prepared and attempted a rescue experiment to construct the infectious viral particles using six rNDV genomes (Figure 1A). All types of infectious rNDVs were recovered and confirmed to detect NDV antigens using immunofluorescence (Additional file 1B). To assess the BRV antigen expression, the HEp-2 cells were infected with each recombinant virus. As shown in Figure 1B, non-infected HEp2 cells (mock) were not stained with either chicken anti-NDV serum or goat anti-BRV antibodies. Cells infected with rNDV-WT, rNDV-WT-VP4, rNDV-2F2HN, and rNDV-2F2HN-VP4 were only detected with chicken anti-NDV serum. rNDV-WT-VP6 and rNDV-2F2HN-VP6 infected cells were detected with anti-BRV antibody and anti-NDV serum. Contrastingly, VP4 protein expression was not detected using anti-BRV antibody (Figure 1B). Similar results were obtained even using the bovine cell lines (MDBK cells) (Additional file 1C). The insertion of VP4 gene into the recombinant viral genome was confirmed using the RT-PCR (Additional File 1D). Therefore, an insufficient VP4 expression was observed in this recombinant NDV system.; Accordingly, we proceeded with the experiments using rNDV-WT-VP6 and rNDV-2F2HN-VP6.

To investigate whether the insertion of the VP6 gene affected the growth properties of the parental NDV strain, we evaluated the growth kinetics of each virus in different cell lines, including the bovine cell lines (BIE and MDBK cells). Both rNDV-WT-VP6 and rNDV-2F2HN-VP6 showed replication kinetics similar to those of the parental vectors (rNDV-WT and rNDV-2F2HN, respectively; Figure 1C). In addition, the genetic stability of the VP6 gene within rNDV-WT-VP6 or rNDV-2F2HN-VP6 was assessed by serially passaging the viruses in MDBK cells five times and confirmed by RT-PCR and immunofluorescence assays (Additional file 2). These results suggested that VP6 gene insertion does not adversely affect NDV replication and that the virus can grow even in bovine cell lines.

### Low pathogenicity of recombinant NDVs expressing BRV-VP6 in poultry and mice

The pathogenicity of rNDV-WT-VP6, rNDV-2F2HN-VP6, and their parental vector viruses was determined in 9-day-old embryonated eggs using an MDT assay. NDV strain with an MDT of more than 90 h is classified as a lentogenic strain, and, notably, all the constructed recombinant viruses showed MDT values of more than 192 h. To assess the safety of these recombinant viruses in mammals, 4-week-old female BALB/c mice were inoculated with 1 × 10^7^ FFU of each virus via the intranasal or intraperitoneal routes (Additional file 3). All mice gained weight during the experiment, with no significant among-group differences. These results indicated that a high titer of rNDV-WT-VP6 and rNDV-2F2HN-VP6 was obtained using the embryonated eggs and that these recombinant vaccine vectors did not show severe pathogenicity in mice.

### VP6-specific antibodies were induced in mice immunized with rNDV-WT-VP6

**Figure 2:**
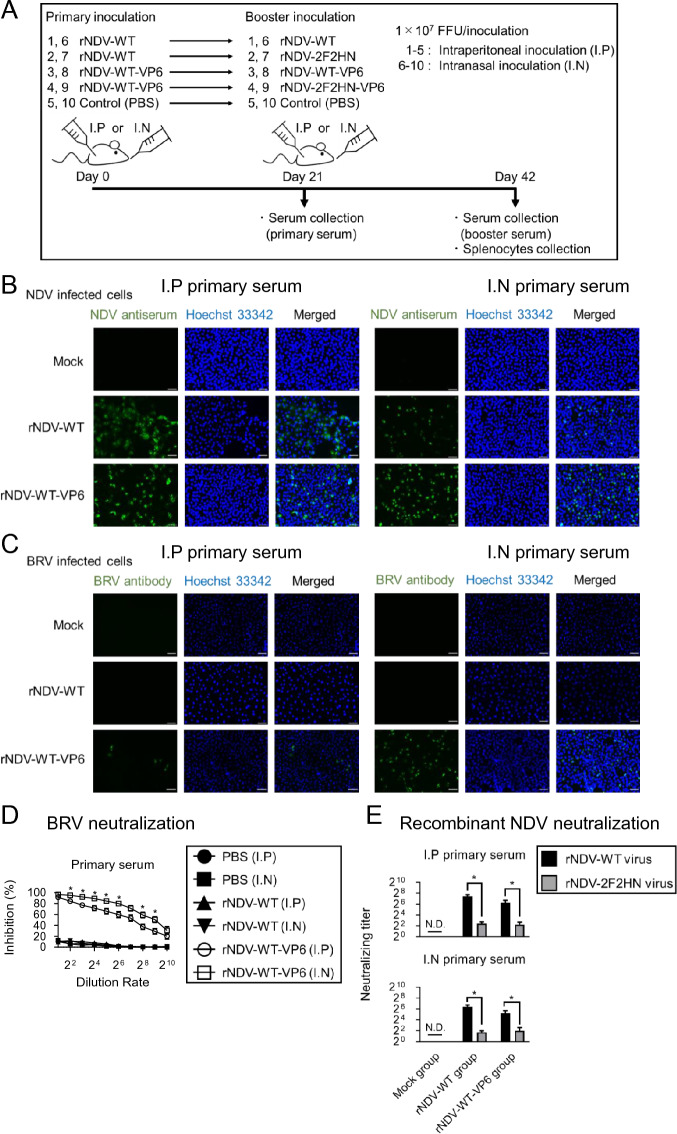
**Primary inoculation of rNDV-WT-VP6 induced specific immune responses against NDV and BRV-VP6 antigens.** **A** Schematic diagram illustrating the immunization schedule and experimental group. The immunogenicity of the recombinant NDVs was evaluated in 4-week-old female BALB/c mice, which were divided into 10 groups (each *n* = 3, five groups for each administration route). The mice in groups 1–5 were inoculated intraperitoneally while mice in groups 6–10 were inoculated intranasally. **B** NDV-infected cells and **C** BRV-infected cells were stained using mouse sera (green) collected after primary inoculation and Hoechst 33342 (blue; nuclei). Scale bar, 50 µm. **D** BRV infection was performed after treatment with each dilution of antiserum. The rate of decrease from 100 FFU is shown as BRV infection inhibition ratio. **E** The maximum dilution ratio for complete NDV neutralization. Data represent the mean ± SEM (*n* = 3, *P < 0.05).

**Figure 3:**
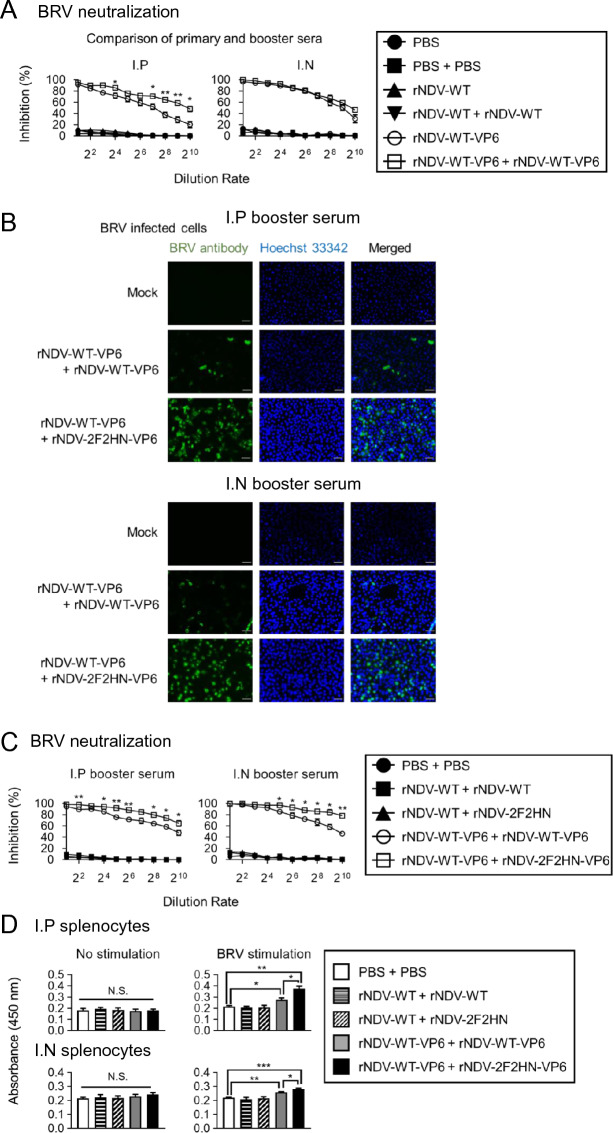
**Combination inoculation elicited higher induction of immune responses compared with homologous booster inoculation.** **A** Comparison of BRV neutralization between primary and booster sera. **B** BRV-infected cells were stained with mouse sera (green) collected after booster inoculation and Hoechst 33342 (blue; nuclei). Scale bar, 50 µm. **C** The BRV infection inhibition ratio was obtained using mouse sera collected after booster inoculation. **D** Assessment of the specific cell proliferation of the mice splenocytes after booster inoculation using MTT assay. (*n* = 3, *P < 0.05, **P < 0.01, ***P < 0.001).

The schedule of the rNDV inoculation experiments is shown in Figure 2A. The effects of primary inoculation (Figures 2B–E) and booster inoculation (Figure 3) were evaluated. To assess the induction of immune responses against the VP6 antigen, the mice were inoculated with 1 × 10^7^ FFU of rNDV-WT or rNDV-WT-VP6 via the intraperitoneal or intranasal route (Figure 2A). After 3 weeks, the mouse sera (primary serum) were obtained. Immunofluorescence analysis revealed that both NDV and BRV antigens were detected by serum collected from mice inoculated with rNDV-WT-VP6 (Figures 2B and C). Notably, the intranasally inoculated group showed higher antigen detection levels than the intraperitoneally inoculated group. Subsequently, we evaluated the neutralizing activity (Figures 2D and E). A neutralization assay was conducted using 100 FFU of BRV to calculate the number of positive cells. Sera obtained from mice inoculated with rNDV-WT-VP6 inhibited the BRV infection in a serum dilution-dependent manner. Consistent with the immunofluorescence assay results (Figure 2B and C), the mice intranasally inoculated with rNDV-WT-VP6 showed a significantly stronger immune response against BRV than the intraperitoneally inoculated mice (Figure 2D). Furthermore, we evaluated the induction of neutralizing antibodies against NDV. Using 1000 FFU of rNDV-WT or rNDV-2F2HN, we investigated the maximum dilution ratio that completely inhibited the infection. Higher neutralizing titers were detected when rNDV-WT, with or without VP6, was used for immunization (Figure 2E). On the contrary, there was weak cross-reactivity with rNDV-2F2HN given the lack of NDV F and HN antigens, as confirmed by a lower neutralizing titer (Figure 2E). Taken together, these results suggested that specific antibodies against VP6 were produced in the mice and that a single inoculation of rNDV-WT-derived viruses elicited high levels of neutralizing antibodies against NDV, regardless of the inoculation route.

### Chimeric rNDV-2F2HN-VP6 booster inoculation induced a higher immune response in mice compared to homologous rNDV-WT-VP6 booster inoculation

Three weeks after the booster inoculation, mouse sera were collected for analysis (Figure 2A). To validate the effects of the booster inoculation, neutralizing activities against BRV were compared between single and booster inoculations using mouse sera inoculated with rNDV-WT-VP6 (Figure 3A). Intraperitoneal inoculation resulted in a booster effect; however, intranasal inoculation did not enhance the neutralizing antibody titer compared with the single inoculation. Subsequently, similar to the primary inoculation (Figure 2), we performed antigen detection in infected cells and compared the inhibition of BRV infection between the rNDV-WT-VP6 and rNDV-2F2HN-VP6 booster inoculation groups. (Figures 3B and C). As shown in Figure 3B, combinational inoculations of rNDV-WT-VP6 priming followed by rNDV-2F2HN-VP6 boosting induced a relatively higher anti-BRV antibody level for both the intraperitoneal and intranasal inoculation groups. The detection rate was correlated with the neutralizing activity within the same inoculation group. Notably, mice that received rNDV-2F2HN-VP6 as a booster inoculation exhibited significantly higher anti-BRV neutralizing antibody levels than those that received a homologous booster inoculation of rNDV-WT-VP6 (Figure 3C).

### Enhanced splenocyte proliferation in response to specific antigens with combination inoculation

To comprehensively evaluate the immune response to BRV, we conducted a splenocyte proliferation assay (Additional file 4C). Splenocytes obtained from mice inoculated with VP6-expressing rNDVs exhibited robust proliferation upon BRV stimulation (Figure 3D). Notably, the cell proliferation was more pronounced with combination booster inoculation than with homologous booster inoculation (Figure 3D).

### Cross-reactivity with another rotavirus strain

We further confirmed that the induced anti-BRV VP6 antibodies exhibited cross-reactivity with a closely related, yet heterologous, Group A rotavirus strain, i.e., the simian rotavirus SA11 strain (Additional file 4A). This cross-reactivity could be attributed to the substantial conservation of VP6 antigenicity among Group A rotaviruses [36–38]. Further, we confirmed neutralizing activity against the simian rotavirus using sera collected from mice inoculated with rNDV-WT-VP6 (Additional files 4B–D). Finally, we confirmed that the simian rotavirus SA11 strain could induce proliferation of mouse splenocytes.

## Discussion

The Group A BRV is a globally distributed virus that causes calf diarrhea and leads to serious problems in newborn calves, including high mortality rates, increased treatment expenses, and impaired growth [39]. Currently, inactivated rotavirus vaccines are administrated to pregnant dams [2]. Compared with live attenuated vaccines, inactivated vaccines are generally less potent at inducing immunity. However, live attenuated vaccines carry the risk of revertance to virulence [8]. Previously, we developed a recombinant chimeric NDV (rNDV-2F2HN) that demonstrated potential as a cancer therapy tool even in the presence of anti-NDV antibodies [26]. The potential of NDV as a vaccine vector has been demonstrated in a range of species, including humans and animals such as cattle, dogs, African green monkeys, rhesus monkeys, pigs, minks, and chickens [40]. In the present study, we generated both wild-type and chimeric rNDVs to express the BRV VP6 antigen and investigated their suitability as vaccine vectors for eliciting strong immunity against BRV in a mouse model. Additionally, we attempted to express another BRV outer capsid protein, VP4, with rNDV-2F2HN. However, VP4 expression was not detected even in bovine cell lines. Previous studies have demonstrated that the expression level of the foreign gene inserted into a full-length clone of NDV is largely affected by the size and sequence of the insert, and the expression of larger-sized genes would be challenging [41, 42]. Accordingly, we measured the expression level of the foreign antigens using flow cytometry (data not shown). It was observed that VP4 protein was expressed, albeit at substantially lower levels compared to the VP6 protein. As a result, there was no detectable level of expression observed via immunostaining, and it was concluded that rNDV-WT-VP4 and rNDV-2F2HN-VP4 were not viable candidates for vaccination due to the lack of VP4 antigenicity. Consequently, our focus turned to rNDV-2F2HN-VP6 as a potential vaccine candidate for BRV.

We demonstrated the efficient propagation of both rNDV-WT-VP6 and rNDV-2F2HN-VP6 in embryonated chicken eggs, which maintained consistently high titers even after multiple passages. These findings highlight the advantages of both recombinant vaccine vectors in yielding high quantities at a low cost, which is consistent with previous reports [43]. To evaluate the safety of rNDV-2F2HN-VP6, we used a mouse model with intraperitoneal and intranasal inoculations. Regardless of the administration route, all mice exhibited post-inoculation weight gain. To assess the immunogenicity of the constructed vaccine candidates, mice were inoculated twice via intranasal or intraperitoneal route. Interestingly, the results of the prime inoculation showed a higher induction of immune responses in the serum of the intranasally inoculated group than in the intraperitoneally inoculated group. This finding is in agreement with a previous work that used rNDV expressing simian immunodeficiency virus antigens for evaluation in mice [44]. They reported that intranasal inoculation induced the strongest immune response among all other routes. This may be attributed to that NDV infects naturally via the respiratory tract and can induce humoral and cellular immune responses both at the mucosal and systemic levels. While earlier reports suggested that antibodies elicited against rotavirus inner capsid VP6 are non-neutralizing in classical neutralization assays [45, 46], our neutralization assay results revealed that VP6-specific antibodies exhibited an inhibitory effect of the BRV infection, varying with serum dilution. Comparable findings were noted in a prior study examining the effects of the VP6 vaccine candidate in calves, where they also detected VP6-specific neutralizing antibodies at lower titers, utilizing the same method as in our investigation [47]. We hypothesized that the potential mechanism involving extracellular neutralization of the virus might have occurred, albeit to a limited extent. However, other potential mechanisms involving VP6 IgG and IgA would also be possible, consisting of intracellular disruption of the viral replication cycle (In classical NT tests, VP6-specific antibodies are not neutralizing) [48]. Although rNDV-WT-VP6 inoculation induced specific immune responses against the VP6 antigen in mice, it also resulted in the production of neutralizing antibodies against NDV. Accordingly, using rNDV-WT-VP6 as a booster did not significantly enhance the immunity induced by the primary inoculation, especially in the intranasally inoculated group. By contrast, the chimeric rNDV-2F2HN-VP6 vaccine vector, in which NDV HN and F proteins were replaced with those of APMV-2, has shown the ability to evade preexisting anti-NDV immunity. The antigenicity of NDV is determined by the surface antigens of F and HN proteins [49]. We have reported in vitro that rNDV-2F2HN escapes neutralization by NDV antiserum [26], and this study indicated that it also escaped in vivo. Therefore, it is considered that the immune system recognized rNDV-2F2HN-VP6 as a new antigen in the booster inoculation. As a result, booster inoculation with rNDV-2F2HN-VP6 induced a significantly enhanced immune response in mice. Furthermore, splenocytes obtained from mice immunized with rNDV-WT-VP6 and rNDV-2F2HN-VP6, but not control rNDVs, showed specific proliferation when stimulated with BRV (Figure 3D). Notably, rNDV-2F2HN-VP6 booster inoculation led to a higher cell proliferation rate than rNDV-WT-VP6 booster inoculation, regardless of the administration route.

Given the high degree of antigenic identity among VP6 antigens of all Group A rotaviruses, eliciting an immune response against VP6 may allow heterotypic protection against other various rotavirus infections [36, 37]. Numerous studies have assessed the intracellular neutralization capacity of VP6 antibodies, revealing their ability to inhibit viral replication in the sera [45, 46, 50]. Therefore, it is believed that VP6 antibodies could play a significant role in alleviating symptoms. In addition, there have been many reports that highlighted the protective impact of VP6 antibodies against rotavirus infection [36, 47, 51–53]. These antibodies have been shown to be effective in several animals, such as calves and mice. Hence, these protective mechanisms are anticipated to be operative across a broad spectrum of animal species. We demonstrated that sera and splenocytes obtained from mice immunized with VP6-expressing rNDVs displayed reactivity with simian rotavirus-infected cells (Additional file 4). These findings indicate the potential utility of VP6-based vaccines against other species of Group A Rotavirus, which concurs with a previous report [52]. Rotaviruses exhibit wide genetic diversity, in particular of the VP7 and VP4 (and NSP1) encoding genes, and possess the ability to reassort and mutate, resulting in the emergence of new strains that can evade preexisting immune responses [54]. Since VP6 proteins are highly immunogenic and possess the remarkable ability to induce potent homologous and cross-reactive immune responses, therefore, a live vaccine vector targeting the BRV VP6 might offer effective protection against other Group A rotaviruses. Our data suggest that the immune cells recognized the chimeric NDV as a novel antigen, which enhanced immune cell proliferation and boosted the immune reactions. Studies using large animals (models) are warranted to elucidate the vaccination efficacy of these recombinant viruses.

### Supplementary Information

**Additional file 1: Confirmation of recombinant NDVs inserted BRV VP4 or VP6.** (**A**) Schema of constructed recombinant NDV genomes inserted with the BRV VP6 or VP4 gene. (**B**) indicates viral titer of the rescued viruses grown in embryonated eggs. (**C**) MDBK cells infected with either rNDV-WT-VP4 or rNDV-2F2HN-VP4 were stained by anti-NDV antiserum (green), BRV antibody (red), and Hoechst 33342 (blue; nuclei). (**D**) Electrophoresis of the PCR products for the verification of BRV-VP6 and VP4 antigens within the constructed viruses using each primer set as indicated. Arrows indicate the expected size (VP6: 1354 bp and VP4: 2511 bp).

**Additional file 2: Assessment of genetic stability of VP6 gene within rNDV-WT-VP6 and rNDV-2F2HN-VP6 viruses.** (**A**) The recovered viruses were serially passaged 5 times in MDBK cells. After the five passages, expression of the VP6 gene was confirmed by immunostaining using anti-BRV antibody. (**B**) VP6 inserted region was amplified. Electrophoresis of PCR products was performed. Arrows indicate the expected size.

**Additional file 3: Pathogenicity of the recombinant viruses in mice.** Weight changes in the mice inoculated intraperitoneally or intranasally with 1 × 10^7^ FFU of each recombinant NDV. The mice in each group (*n* = 3) were observed and weighed daily for 14 days.

**Additional file 4: Cross-reactivity of mice serum antibodies to SA11 Rotavirus.** (**A**) SA11 rotavirus-infected cells were stained by mice serum (green) after primary inoculation and Hoechst 33342 (blue; nuclei). (**B**) indicates SA11 rotavirus infection inhibition ratio using mice serum collected after primary inoculation. (**C**) Schematic drawing illustrating specific cell proliferation assay of mice splenocytes. (**D**) Assessment of specific cell proliferation of mice splenocytes after booster inoculation using MTT assay. (*n* = 3, *P < 0.05, **P < 0.01, ***P < 0.001).

**Additional file 5: Primer sets used in this study.**

**Additional file 6: Schema of the base sites for each primer set.**

## Data Availability

All data generated or analyzed during this study are included in this published article and its supplementary information files.
