# Supplementary figures and images for: Use of live attenuated recombinant Newcastle disease virus carrying avian paramyxovirus 2 HN and F protein genes to enhance immune responses against species A rotavirus VP6 protein
Source: Vet Res. 2024 Feb 5;55:16. doi: 10.1186/s13567-024-01271-4 (PMC10845738; doi:10.1186/s13567-024-01271-4)

## Slide 1
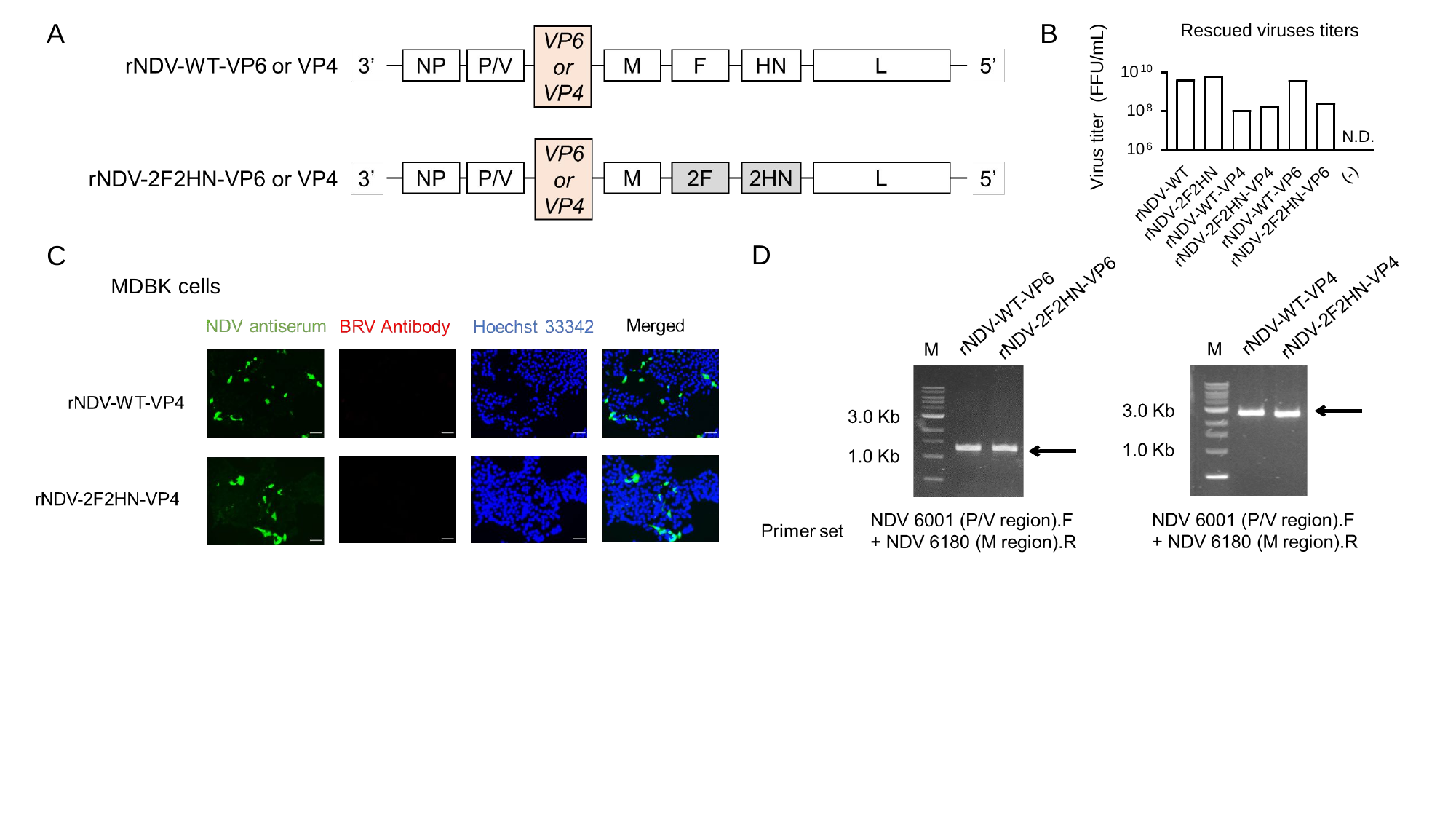

A
B
D
C
MDBK cells

Supplement: Supplementary file 1 — Additional file 1: Confirmation of recombinant NDVs inserted BRV VP4 or VP6. (A) Schema of constructed recombinant NDV genomes inserted with the BRV VP6 or VP4 gene. (B) indicates viral titer of the rescued viruses grown in embryonated eggs. (C) MDBK cells infected with either rNDV-WT-VP4 or rNDV-2F2HN-VP4 were stained by anti-NDV antiserum (green), BRV antibody (red), and Hoechst 33342 (blue; nuclei). (D) Electrophoresis of the PCR products for the verification of BRV-VP6 and VP4 antigens within the constructed viruses using each primer set as indicated. Arrows indicate the expected size (VP6: 1354 bp and VP4: 2511 bp). [file 13567_2024_1271_MOESM1_ESM.pptx]

## Slide 1
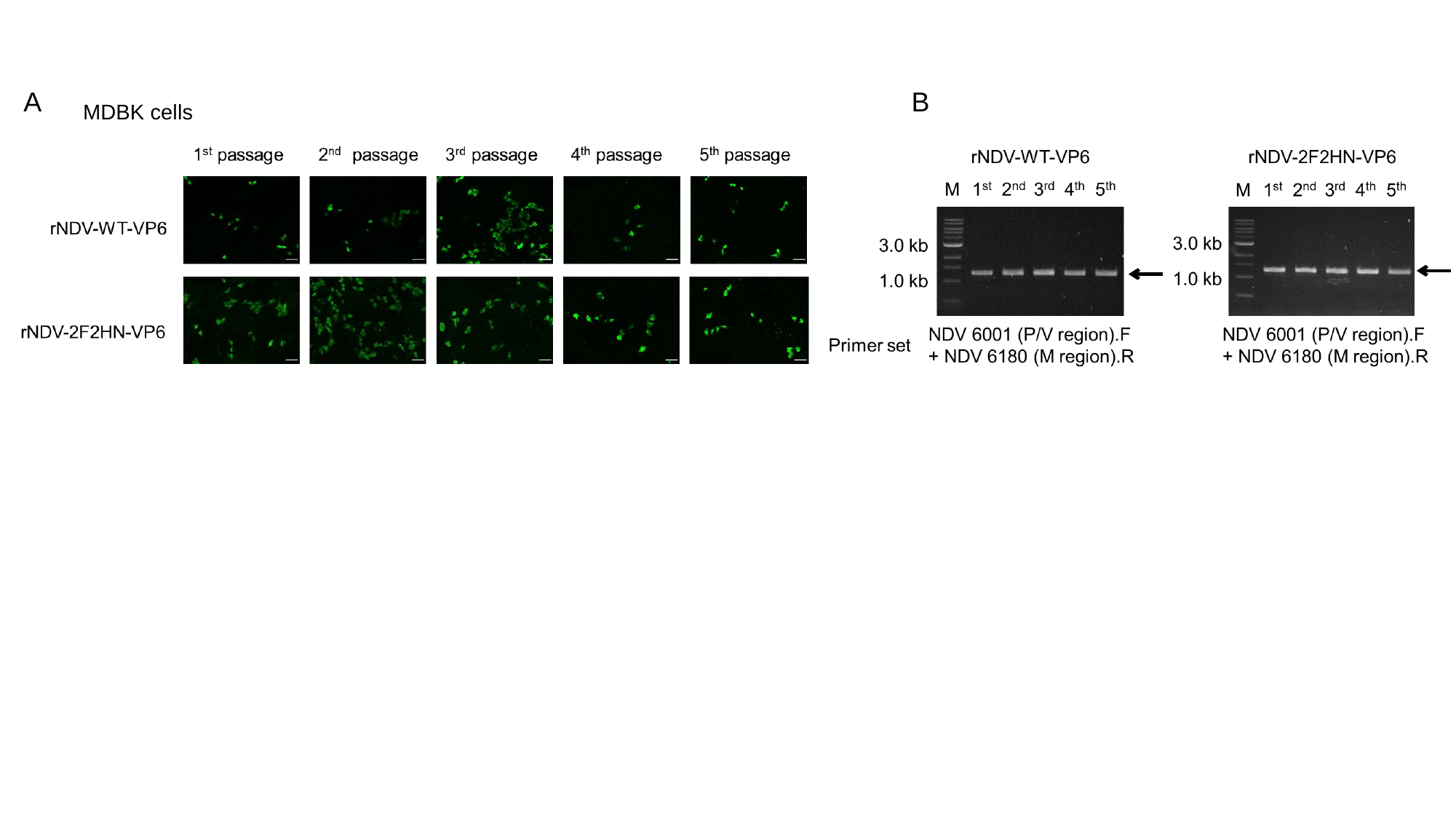

A
B
MDBK cells

Supplement: Supplementary file 2 — Additional file 2: Assessment of genetic stability of VP6 gene within rNDV-WT-VP6 and rNDV-2F2HN-VP6 viruses. (A) The recovered viruses were serially passaged 5 times in MDBK cells. After the five passages, expression of the VP6 gene was confirmed by immunostaining using anti-BRV antibody. (B) VP6 inserted region was amplified. Electrophoresis of PCR products was performed. Arrows indicate the expected size. [file 13567_2024_1271_MOESM2_ESM.pptx]

## Slide 1
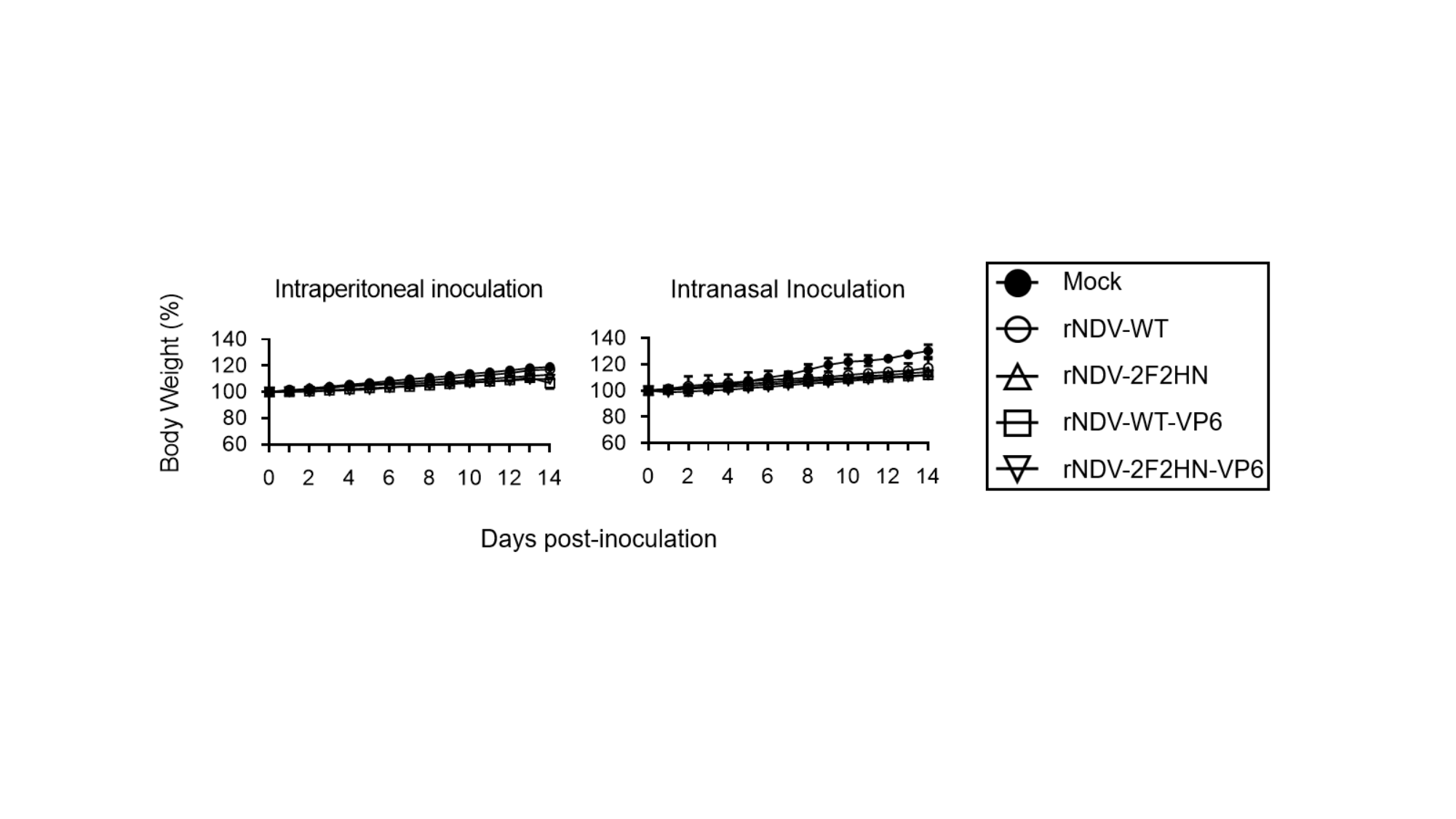

Supplement: Supplementary file 3 — Additional file 3: Pathogenicity of the recombinant viruses in mice. Weight changes in the mice inoculated intraperitoneally or intranasally with 1 × 10^7^ FFU of each recombinant NDV. The mice in each group (n = 3) were observed and weighed daily for 14 days. [file 13567_2024_1271_MOESM3_ESM.pptx]

## Slide 1
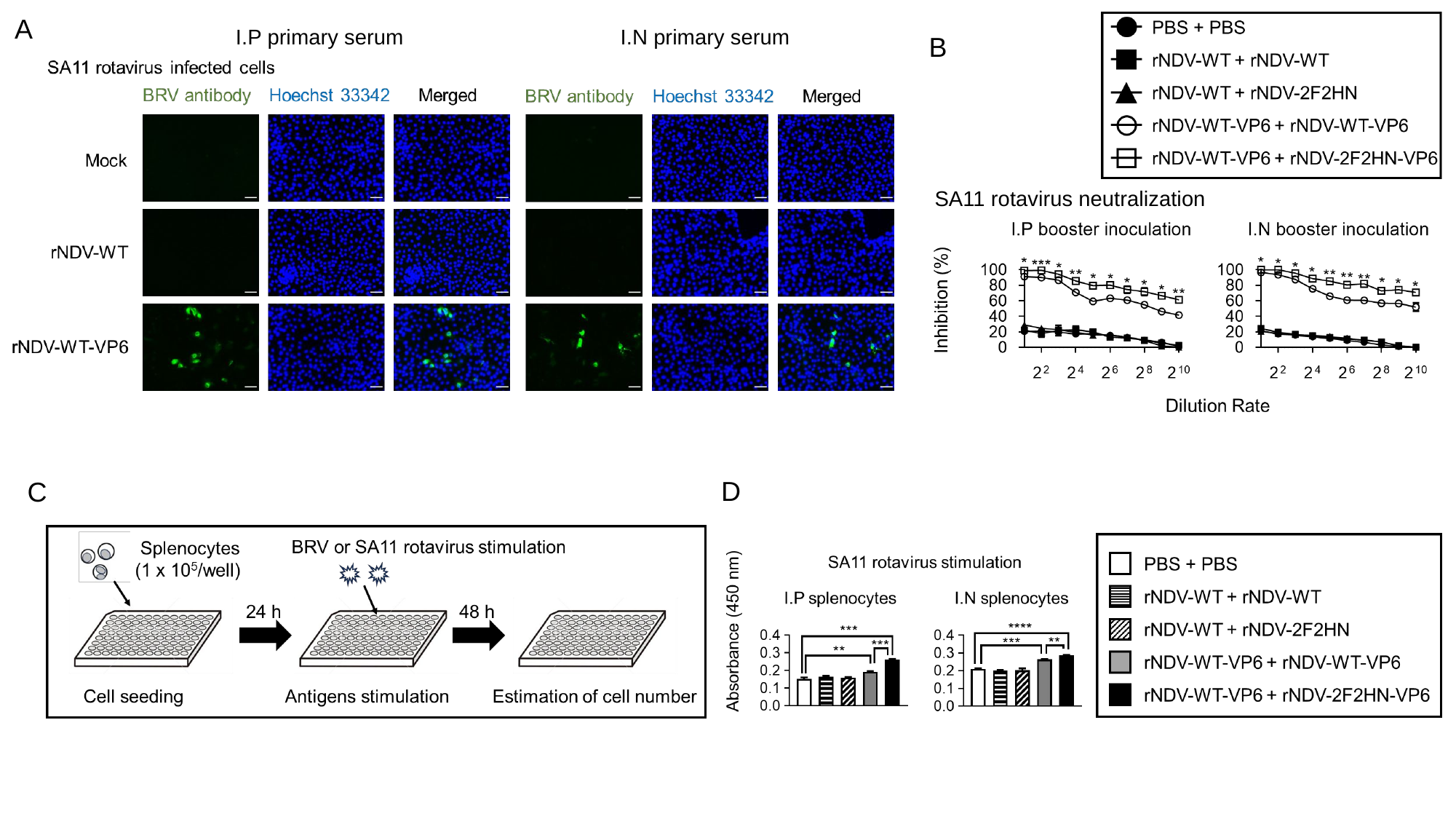

A
I.P primary serum
I.N primary serum
B
SA11 rotavirus neutralization
D
C

Supplement: Supplementary file 4 — Additional file 4: Cross-reactivity of mice serum antibodies to SA11 Rotavirus. (A) SA11 rotavirus-infected cells were stained by mice serum (green) after primary inoculation and Hoechst 33342 (blue; nuclei). (B) indicates SA11 rotavirus infection inhibition ratio using mice serum collected after primary inoculation. (C) Schematic drawing illustrating specific cell proliferation assay of mice splenocytes. (D) Assessment of specific cell proliferation of mice splenocytes after booster inoculation using MTT assay. (n = 3, *P < 0.05, **P < 0.01, ***P < 0.001). [file 13567_2024_1271_MOESM4_ESM.pptx]
