# Supplementary material for: Use of live attenuated recombinant Newcastle disease virus carrying avian paramyxovirus 2 HN and F protein genes to enhance immune responses against species A rotavirus VP6 protein
Source: Vet Res. 2024 Feb 5;55:16. doi: 10.1186/s13567-024-01271-4 (PMC10845738; doi:10.1186/s13567-024-01271-4)
Supplement: Supplementary file 5 — Additional file 5: Primer sets used in this study. [file 13567_2024_1271_MOESM5_ESM.pptx]

## Slide 1
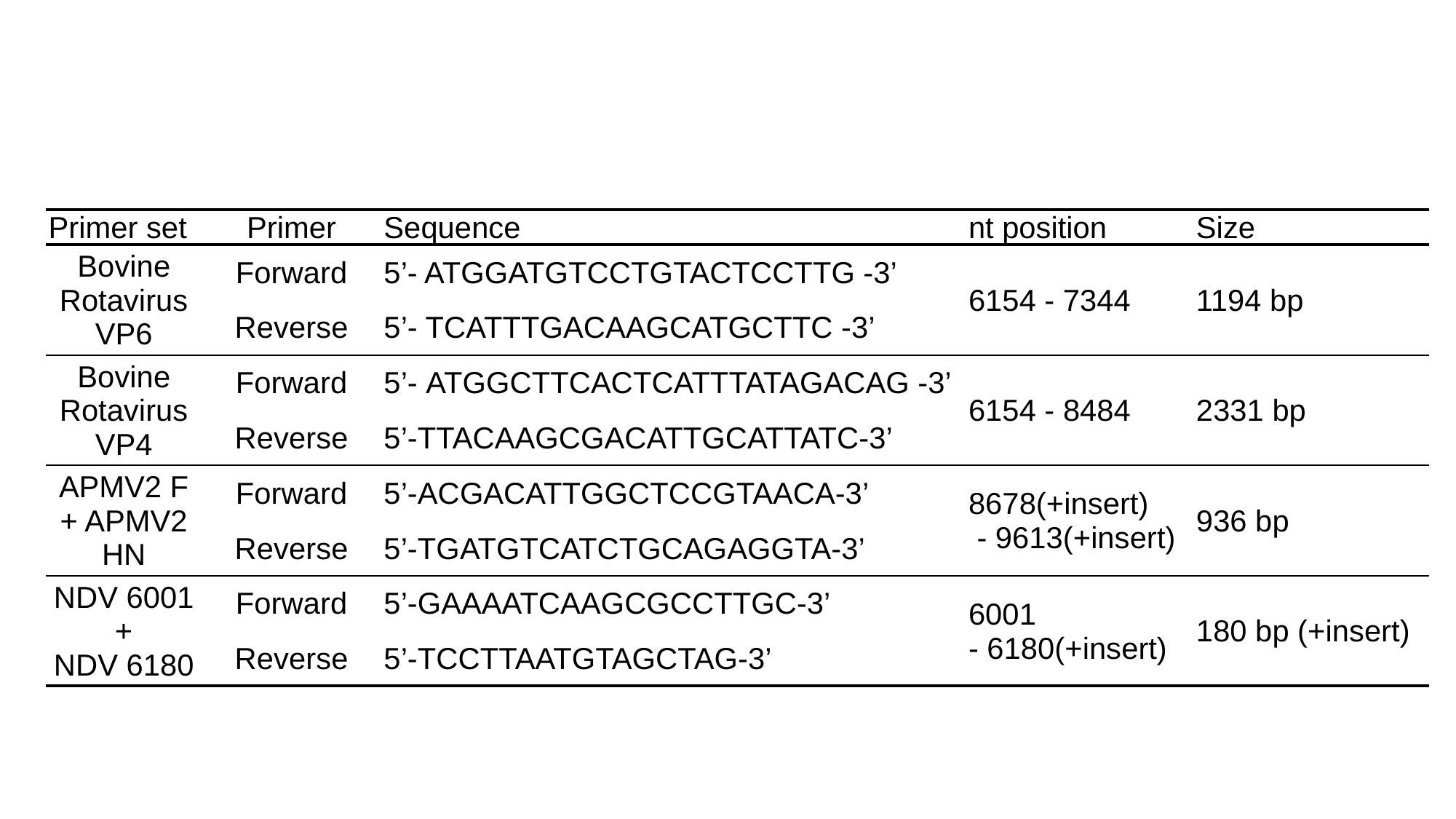

| Primer set | Primer | Sequence | nt position | Size |
| --- | --- | --- | --- | --- |
| Bovine Rotavirus VP6 | Forward | 5’- ATGGATGTCCTGTACTCCTTG -3’ | 6154 - 7344 | 1194 bp |
| | Reverse | 5’- TCATTTGACAAGCATGCTTC -3’ | | |
| Bovine Rotavirus VP4 | Forward | 5’- ATGGCTTCACTCATTTATAGACAG -3’ | 6154 - 8484 | 2331 bp |
| | Reverse | 5’-TTACAAGCGACATTGCATTATC-3’ | | |
| APMV2 F + APMV2 HN | Forward | 5’-ACGACATTGGCTCCGTAACA-3’ | 8678(+insert) - 9613(+insert) | 936 bp |
| | Reverse | 5’-TGATGTCATCTGCAGAGGTA-3’ | | |
| NDV 6001 + NDV 6180 | Forward | 5’-GAAAATCAAGCGCCTTGC-3’ | 6001 - 6180(+insert) | 180 bp (+insert) |
| | Reverse | 5’-TCCTTAATGTAGCTAG-3’ | - 6180 | |
