# Supplementary material for: Use of live attenuated recombinant Newcastle disease virus carrying avian paramyxovirus 2 HN and F protein genes to enhance immune responses against species A rotavirus VP6 protein
Source: Vet Res. 2024 Feb 5;55:16. doi: 10.1186/s13567-024-01271-4 (PMC10845738; doi:10.1186/s13567-024-01271-4)
Supplement: Supplementary file 6 — Additional file 6: Schema of the base sites for each primer set. [file 13567_2024_1271_MOESM6_ESM.pptx]

## Slide 1
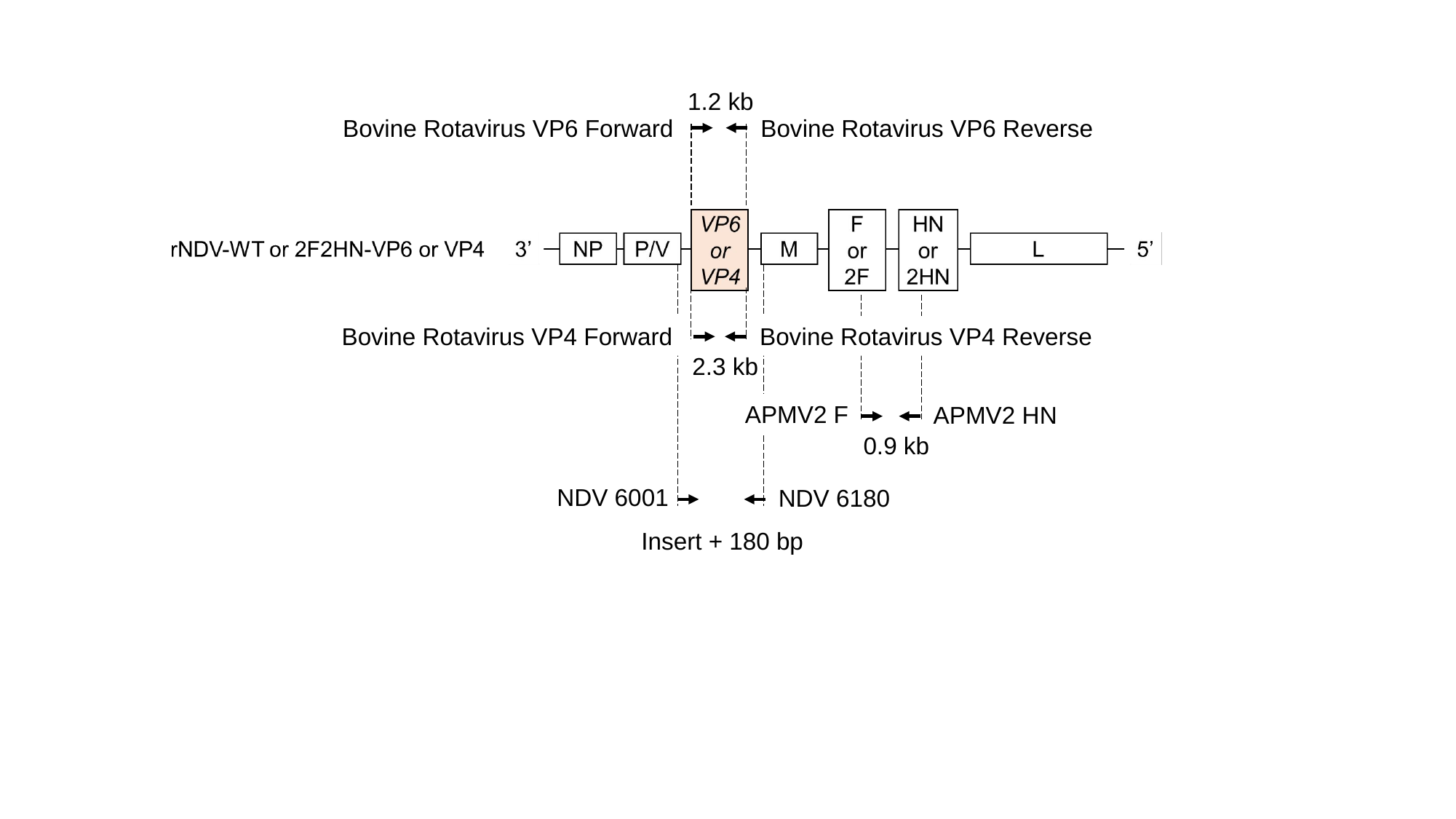

1.2 kb
Bovine Rotavirus VP6 Forward
Bovine Rotavirus VP6 Reverse
Bovine Rotavirus VP4 Forward
Bovine Rotavirus VP4 Reverse
2.3 kb
APMV2 F
APMV2 HN
0.9 kb
NDV 6001
NDV 6180
Insert + 180 bp
